# Choriocapillaris changes in dry age-related macular degeneration and geographic atrophy: a review

**DOI:** 10.1186/s40662-018-0118-x

**Published:** 2018-09-15

**Authors:** Malvika Arya, Almyr S. Sabrosa, Jay S. Duker, Nadia K. Waheed

**Affiliations:** 1 New England Eye Center, Tufts Medical Center, Boston, MA USA; 2 Institude of Ophthalmology, Rio de Janeiro, Brazil

**Keywords:** Dry age-related macular degeneration, Geographic atrophy, Choriocapillaris, Optical coherence tomography, Optical coherence tomography angiography

## Abstract

Age-related macular degeneration (AMD) is a leading cause of central vision loss worldwide. The progression of dry AMD from early to intermediate stages is primarily characterized by increasing drusen formation and adverse impact on outer retinal cells. Late stage AMD consists of either geographic atrophy (GA), the non-exudative (dry) AMD subtype, or choroidal neovascularization, the exudative (wet) AMD subtype. GA is characterized by outer retinal and choroidal atrophy, specifically the photoreceptor layer, RPE, and choriocapillaris. Much remains to be discovered regarding the pathogenesis of AMD progression and subsequent development of GA. As the functionality of all three layers is closely linked, the temporal sequence of events that end up in atrophy is important in the understanding of the pathogenic pathway of the disease. The advent of OCTA, and particularly of swept-source technology, has allowed for depth-resolved imaging of retinal vasculature and the choriocapillaris. With the use of OCTA, recent studies demonstrate that choriocapillaris flow alterations are closely associated with the development and progression of AMD. Such changes may even possibly offer predictive value in determining progression of GA. This article reviews studies demonstrating choriocapillaris changes in dry AMD and summarizes the existing literature on the potential role of the choriocapillaris as a key factor in the pathogenesis of AMD.

## Background

The prevalence of age-related macular degeneration (AMD), currently at 6.5% [1] in the US population aged 40 years and above, continues to expand, and is projected to globally affect 196 million people by 2020 [2]. In the US, AMD accounts for more than 54% of visual loss amongst the Caucasian population[3]. The widespread nature of dry AMD [4, 5] and the unpredictability of its progression to choroidal neovascularization (CNV), geographic atrophy (GA), or both with sight threatening implications, continues to draw the interest of many investigators to better understand its pathogenesis. The association of dry AMD with the late AMD stages of CNV and GA has intrigued various researchers, and a possible underlying “unified” vascular abnormality has been suggested in its pathogenesis [6–9]. Advances in multimodal imaging have enhanced our understanding of dry AMD, including the identification of high risk features for its progression to GA and CNV, by facilitating high quality in vivo imaging. OCT angiography (OCTA), as a non-invasive, depth-resolved imaging modality has allowed us to explore the role of choroidal vasculature in the pathogenesis of AMD [10–12].

### Classification of dry AMD and geographic atrophy

The classification of AMD provides a framework to assess severity in a clinical setting and to gauge the efficacy of therapy. Various AMD grading systems are based on color fundus photography and applied in a clinical setting. Seddon et al. used the Clinical Age-Related Maculopathy Staging system [13] to categorize AMD into the following stages: grade 1 as no AMD (no drusen or a few drusen < 63 μm), grade 2 as early AMD (intermediate-size drusen 63–124 μm), grade 3 as intermediate AMD (large drusen ≥125 μm), grade 4 as geographic atrophy with or without foveal involvement, and grade 5 with neovascularization. However, color fundus photographs are limited in their identification of certain features of dry AMD, such as subretinal drusenoid deposits (SDD) and morphological alterations in RPE adjacent to GA, thereby generating inaccuracies in their classification of AMD. The wide spectrum of phenotypic variations of GA [14] facilitated the development of an OCT-based classification system of GA. OCT imaging of GA closely resembles its histopathological characteristics, as described by Sarks et al. on electron microscopic studies [15], and may be helpful in early recognition, allowing for the modification of high risk characteristics in early stages of the disease. On OCT, classical GA is characterized by atrophy of the outer nuclear layer, external limiting membrane (ELM), ellipsoid zone (EZ), photoreceptors, retinal pigment epithelium (RPE), and choriocapillaris (CC), in the setting of characteristic extracellular deposits, causing increased transmission of the OCT signal below Bruch’s membrane [16]. A consensus terminology has been proposed for staging retinal atrophy as complete RPE and outer retinal atrophy (cRORA), incomplete RPE and outer retinal atrophy (iRORA), complete outer retinal atrophy, and incomplete outer retinal atrophy, based on OCT findings [17]. The term nascent GA refers to iRORA, as diminishment of the outer plexiform layer (OPL) and inner nuclear layer (INL) and a break in the ELM, with or without the presence of a hyper-reflective band within the OPL during OCT imaging [16]. Nascent GA was identified as a form of intermediate AMD with high-risk characteristics for progression to GA (cRORA) [16].

The role of the choriocapillaris in the pathogenesis of AMD remains controversial. We performed a review of histopathological and OCT/OCTA imaging studies to explore the role of the choriocapillaris (CC) in the pathogenesis of dry AMD. A literature search was performed on PubMed using various forms of the following keywords: choriocapillaris, dry AMD, geographic atrophy, OCT, OCTA. We also reviewed pertinent articles from the bibliography of citations retrieved during this literature search.

## Review

### Histopathological findings of dry AMD

The RPE acts as a blood-retina barrier and has multiple functions, including the nourishment of photoreceptors, phagocytosis of photoreceptor debris, and wound healing in a symbiotic relationship with its underlying Bruch’s membrane and CC. Choroidal vasculature lacks autoregulation and its hypoperfusion impairs functionality of the RPE and photoreceptor layer. A genetic predisposition, aging, oxidative damage, and inflammation can also disrupt this mutualistic relationship between the RPE, Bruch’s membrane, and CC, participating in the development of drusen and pigmentary abnormalities at the level of the RPE [18].

Various histopathologic studies have implicated CC loss as an initiating factor for the development of AMD, while other investigators have observed a secondary attenuation of the CC triggered by RPE abnormalities. Histopathologic studies have demonstrated that the loss of the CC precedes RPE degeneration [19, 20]. Biesemeier et al. used light and electron microscopy to demonstrate a thickened Bruch’s membrane with increased basal laminar deposits between the RPE and its basement membrane, and basal linear deposits within Bruch’s membrane itself. This was accompanied by an increased loss of photoreceptors, RPE cells, and CC in eyes with AMD. They concluded that CC loss is an aging phenomenon that precedes RPE atrophy and the loss of photoreceptors in AMD [19]. Lengyel et al. demonstrated a spatial relationship between equatorial drusen and intercapillary pillars of the CC, suggestive as an initial site of drusen deposition [21]. Increased sub-RPE deposit density has also been correlated with CC loss and the development of drusen over areas of the choroid with ghost vessels [22]. Pilgrim et al. demonstrated, using a primary cell culture model, that sub-RPE deposits in AMD are produced by the RPE and regulated by a combination of the RPE, loss of permeability of Bruch’s membrane and the CC complex [23]. Seddon et al. studied histopathological changes in the CC in AMD in a small number of eyes, but found that CC loss occurs without RPE atrophy in the early and intermediate stages of AMD [24]. Furthermore, a severe attenuation of the CC was also evident in the submacular area in the later stages of both exudative and non-exudative AMD [24].

Conversely, Seddon et al. also reported RPE atrophy with a preserved choriocapillaris at the edges of GA [24]. A loss of RPE preceding CC atrophy in GA has been well documented [25]. Bhutto and Lutty, following a comprehensive literature review, postulated that in exudative AMD, disruption of the photoreceptor/RPE/Bruch’s membrane/choroidal vascular complex results from an initial insult to choroidal vasculature, whereas RPE dysfunction as a primary insult is predictive of atrophic AMD [18]. They also demonstrated, histopathologically, that preservation of the CC at the edge of RPE atrophy precedes CC attenuation in GA [26]. In areas of total RPE atrophy in GA eyes, McLeod et al. demonstrated a reduction in the mean vascular area of the CC and compromise in its function, without obliteration of the CC [27]. They suggested that RPE degeneration is the primary abnormality due to cellular stress and genetic factors in eyes with GA with secondary choriocapillaris sclerosis [27]. Sarks et al. traced the evolution of GA with clinicopathological studies using electron microscopy [15]. They demonstrated progressive failure of the cellular metabolism of RPE cells with accumulation of basal laminar deposits and shedding of membranous debris. The RPE was attenuated with cellular dysmorphia and layering of abnormal cells in the junctional zone. Age-related patchy thickening and hyalinization of Bruch’s membrane extending to the intercapillary pillars expanded corresponding to the area of incipient atrophy and appeared to result from RPE degeneration [15]. Advanced RPE phenotypic variations and the aggregation of morphologically altered RPE cells adjacent to GA has been demonstrated by other investigators as well [28, 29]. Furthermore, Kochounian et al. found a variant of a retinal G protein-coupled receptor (RGR-d) synthesized by the RPE that is predominantly located at the intercapillary pillars of the CC and precedes the formation of drusen at that location [30]. Bird et al. found loss of photoreceptor cells beyond the edge of GA by light, electronic, and autofluorescence microscopy, in the absence of demonstrable morphological changes of the RPE and Bruch’s membrane [31]. Overall, it remains challenging to differentiate the cause-and-consequence relationship between the surrogate markers of AMD, including drusen deposition and RPE pigmentary changes, and CC loss by histopathological studies. Histopathological assessment lacks the advantage of in vivo cross-sectional imaging of the chorioretinal layers by OCT/OCTA, and longitudinal follow-up of patients to better understand the cause-and-consequence relationship of different tissue changes.

Studies have suggested that the alternative complement pathway and membrane attack complex (MAC) in the choroid also play key roles in the pathogenesis of AMD and GA [32]. Mullins et al. postulated that the deposition of complement pathway complexes acts as an activating event for the loss of the CC in early AMD and for drusen formation [22]. This group also found that eyes with a high risk genotype accompanied by complement gene polymorphism have elevated levels of MAC with an increased risk of CC loss, as compared to eyes with a low risk genotype [33]. The deposition of MAC was observed in the outer aspect of Bruch’s membrane and extracellular matrix of the CC prior to CC loss in early AMD and GA [33]. Chirco et al. also observed the preferential deposition of MAC in the basement membrane of the CC endothelium [34], and choroidal endothelial cells were found to be susceptible to complement-mediated cytolysis following exposure to MAC [35]. Whitmore et al. also described CC loss in the early stages of AMD caused by complement activation [36]. Evidently, molecular mechanisms involved in the pathogenesis of AMD cause inflammation and cellular injury at the level of the CC, resulting in its atrophy.

Studies have shown an increased risk of sub-RPE deposits in dry AMD [37] and of CNV development with smoking [38, 39]. Oxidative radicals, such as hydroquinone, accumulate in Bruch’s membrane and oxidative injury may trigger apoptosis of RPE cells over a period of time [27]. The association of exudative AMD with hypertension [38, 40–42] and incident myocardial infarction [43] is well-documented. However, the specific relationship between hypertension and dry AMD has not yet been identified [40]. The pathogenesis of atherosclerosis is multifactorial and partially guided by inflammatory factors, such as C-reactive protein, lipoprotein(a), fibrinogen, interlukin-6, and complements 3 and 4 [18]. Bhutto and Lutty have demonstrated an increased concentration of lipoprotein(a) in the choroidal arteries of eyes with early wet AMD, suggestive of local inflammatory insult and a possible role of atherosclerosis in the pathogenesis of CNV [18]. Van Leeuwen et al. demonstrated that hypertension and atherosclerosis are independent risk factors for AMD [44].

### OCT/OCTA imaging of dry AMD

In vivo imaging by OCT has significantly enhanced our understanding of chorioretinal disorders, including AMD, with respect to their early recognition, pathogenesis, disease progression, and treatment paradigms. OCT is a non-invasive imaging technique that can generate cross-sectional images at a given retinal location within seconds. OCTA is a functional extension of OCT, and couples angiographic information with the structural information of OCT. Two widely used OCTA types are spectral-domain OCTA (SD-OCTA) and swept-source OCTA (SS-OCTA), which differ primarily in their light source. SD-OCTA consists of a broad-bandwidth light source coupled with a spectrometer, while SS-OCTA uses an array of photodetectors and a tunable laser light source that sweeps through a range of frequencies. Since SS-OCTA systems are not limited by camera reading rates, SS-OCTA can achieve faster acquisition speeds, at 100,000–400,000 A-scans per second. Comparatively, SD-OCTA imaging speeds are ~ 70,000 A-scans per second. Faster scan times allow for a greater retinal field of view and higher resolution due to increased sampling density. Additionally, the light source of SS-OCTA operates at a wavelength of ~ 1050 nm, compared to the 840 nm of SD-OCTA, allowing for increased signal depth-penetration through the RPE, pigmentary clumps, and drusen. This aspect of SS-OCTA is particularly useful in the imaging of the choriocapillaris [45]. Compared to structural OCT, OCTA imaging of the CC has allowed for its better visualization and differentiation [46].

**Fig. 1:**
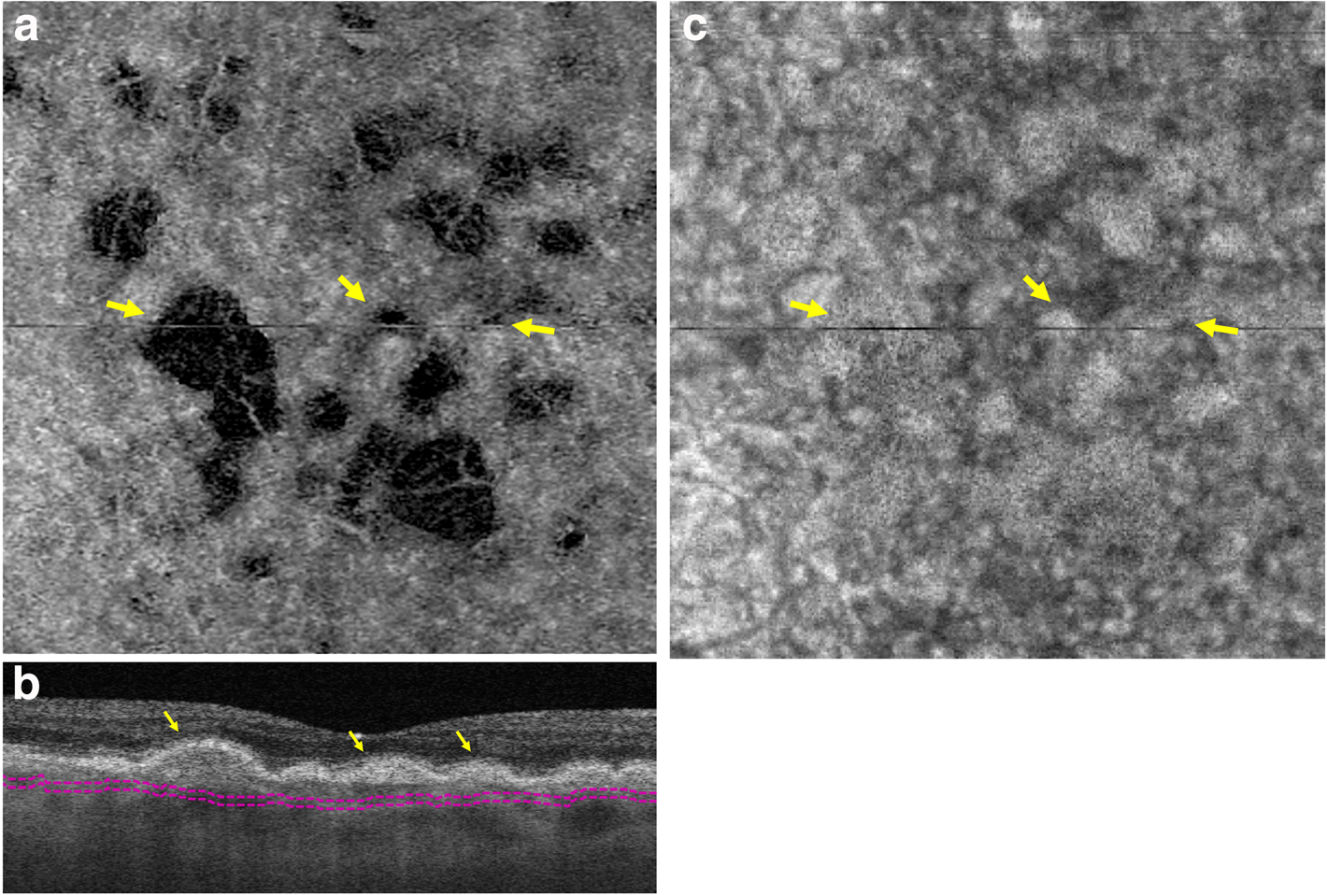
Decreased CC flow under drusen as imaged by swept-source OCTA. En face OCTA of the choriocapillaris (**a**) with corresponding OCT B-scan (**b**) shows areas of decreased flow corresponding to areas beneath drusen (arrows). En face structural OCT (**c**) shows adequate OCT signal penetration

Currently, OCTA imaging is limited in its detection of blood flow in individual choriocapillary vessels. However, areas of absent flow signal (flow voids) are detectable in the CC [47]. Spaide examined these flow voids by OCTA and detected CC flow alterations in the fellow eyes of patients with late AMD [47]**.** Similarly, increased CC void size has been observed in patients with intermediate dry AMD with exudative AMD in the fellow eye, as compared to eyes with intermediate dry AMD without neovascular AMD in the fellow eye [48]. Additionally, SS-OCTA was used to observe a reduction of CC density and focal areas of CC flow impairment in eyes with early dry AMD, as compared to age-matched normal eyes [49]. Figure 1 demonstrates visualization of CC loss using SS-OCTA. A direct correlation between decreasing CC vascular density and increasing density of sub-RPE deposits has also been observed. Subretinal drusenoid deposits (SDD), preferentially located in the rod-rich perifoveal area, and basal linear deposits (BlinD) in the cone-dominant fovea are increasingly prevalent in dry AMD [50]**.** Reticular pseudodrusen (RPD) are also considered SDD by some investigators as precursors of AMD progression [51]. Reduced CC flow and CC vessel density have been observed with RPD [52]. In fact, RPD are associated with greater CC loss as compared to eyes with other drusen [53]. Furthermore, it has been shown that, in a third of patients with RPD, this CC flow impairment extends beyond the margins of the area of RPD itself [54]. All these findings suggest that the accumulation of SDD (RPD) could be a surrogate marker for outer retinal hypoxia. Overall, CC flow alterations appear to play a dominant role in the pathogenesis of dry AMD.

**Fig. 2:**
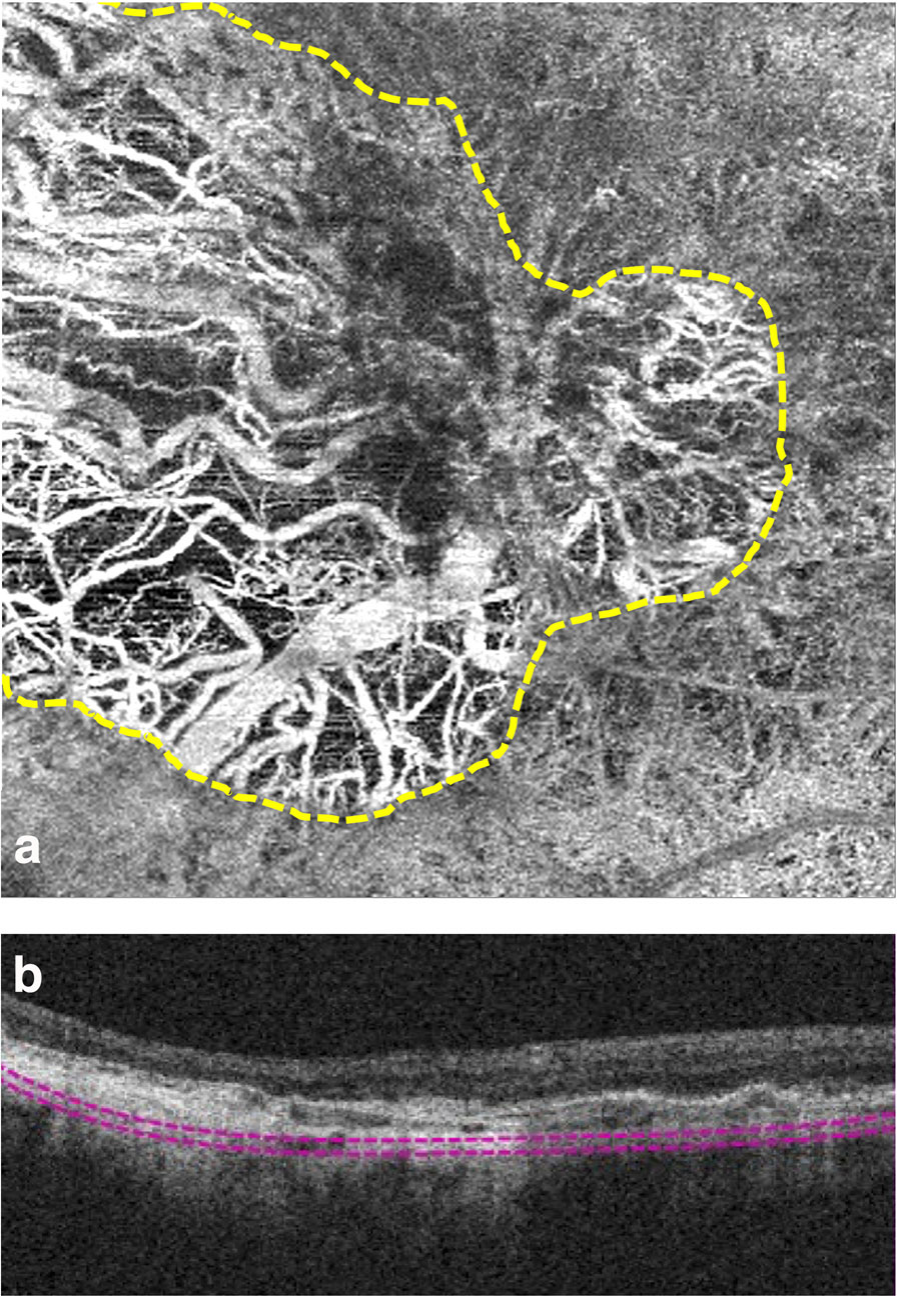
Visualization of geographic atrophy as imaged by spectral-domain OCTA. En face OCTA image (**a**) of the choriocapillaris with corresponding structural OCT B-scan through the lesion (**b**) depicts a well-defined region of geographic atrophy with loss of choriocapillaris within the lesion, allowing for visualization of larger deeper choroidal vessels

OCTA devices are limited in their sensitivity thresholds for the slowest detectable and fastest distinguishable blood flow speeds. Since the decorrelation signal that identifies blood flow is dependent on the interscan time between two consecutive B-scans at a particular location, prolongation of this interscan time would increase the threshold for slowest detectable flow, thereby allowing for distinction between CC atrophy and CC flow attenuation. Variable interscan time analysis (VISTA) alters this interscan time by comparing not only consecutive B-scans, but alternate B-scans as well [55]. Moult et al. used VISTA to observe a slowing of CC flow underneath lesions of nascent GA and CC loss under drusen associated GA [56]. In these areas of CC atrophy, underlying larger choroidal vessels may be displaced upwards and visualized on en face OCTA at the level of the CC [49]. Figure 2 depicts a GA lesion with CC loss and inward displacement of larger choroidal vessels.

CC alterations have also been visualized extending beyond the borders of GA [55]. However, it was noted that CC alterations within the GA lesion itself were primarily atrophic, with substantial flow impairment as well, while those extending beyond the lesion were primarily diminished flow. Asymmetric CC flow impairment of varying degrees was seen beyond the margins of GA [49]. Kavnata et al. also showed CC flow impairment beyond the area of GA, with RPE preservation [57]. Similarly, Sacconi et al. also reported decreased CC vessel density at the margins of GA [58], and Cicinelli et al. found CC diminution, instead of CC loss, in areas bordering GA [59]. Consequently, it has been hypothesized that CC flow impairment at the margins of GA may predict direction and rate of growth of GA [60]. Overall, the aforementioned studies suggest that CC flow alterations may precede overlying RPE atrophy. Perhaps such CC alterations may eventually be able to predict areas of GA progression.

On the contrary, Seddon et al. used OCT to demonstrate that the progression of AMD to NV and GA followed abnormalities at the level of the photoreceptor layer, and particularly the EZ [61]. Pelligrini et al. compared CC impairment in GA to that in Stargardt’s disease [62], characterized by a loss of photoreceptors. Patients with Stargardt’s disease showed an extensive loss of CC with preserved RPE at the margins, whereas GA was primarily associated with RPE loss, leading to photoreceptor loss, and decreased CC circulation at the margins of GA [62]. Thus, these studies suggest that perhaps, the CC, while playing a key role in GA progression, may in fact not be the instigating event itself.

### Limitations of OCT/OCTA

OCT and OCTA have been instrumental in furthering our understanding of the various stages of dry AMD. However, OCTA does come with certain limitations, especially during investigation of the CC. OCTA images may be affected by segmentation errors and various artifacts. Dry AMD causes alterations in the structure of the RPE, such as elevation with drusen, or hyper-transmission of signal with GA. These changes in contour can alter automated segmentation algorithms, thereby affecting qualitative analysis of OCTA en face images or quantitative analysis, such as measurement of the thickness of various choroidal layers. OCTA images can also be affected by projection artifacts, in which overlying vessels appear in deeper retinal layers. These artifacts may particularly affect analysis of the outer retinal and choroidal layers, where flow may be falsely perceived, i.e. in flow void areas. Assessment of the CC may also be limited by shadow artifact. Due to the hyper-reflective nature of the RPE or drusen, signal loss below the RPE or drusenoid deposits may occur, limiting visualization of the CC. These areas of decreased OCTA signal may falsely appear as areas of nonperfusion.

Signal attenuation and segmentation errors in CC slabs in the presence of soft drusen have been well documented as causing false interpretation of CC flow impairment [63]. Lane et al. demonstrated that SS-OCTA, with a longer wavelength of 1050 nm compared to SD-OCTA, was less prone to shadowing artifact in the presence of drusen [45]. Compared to SD-OCTA, SS-OCTA offers increased depth-penetration because of its longer wavelength, and thus, more reliable CC imaging [49]. Despite its limitations, compared to other imaging methods, Corbelli et al. found OCTA to be a reliable modality for the qualitative and quantitative analysis of GA, as well as pathological foveal involvement, and the detection of occult CNV in the presence of dry AMD [64]. They also found manual segmentation to be necessary to overcome segmentation errors, allowing for improved visualization of CC alterations.

Alten et al. demonstrated reliable automated segmentation of early and intermediate AMD by OCTA [63]. However, the reliability of automatic segmentation in the presence of large drusen and GA remains questionable [65]. Moult et al. showed that choosing a slab slightly posterior to the anatomically correct location of the CC may provide more reliable imaging of the CC, due to a persistence of the decorrelation signal posteriorly [66]. Additionally, as described above, the VISTA algorithm has been used to distinguish between CC flow impairment and CC atropthy [56]. To further overcome the limitations of OCTA artifacts, Campbell et al. have described a projection artifact removal algorithm that neutralizes projection artifacts while preserving flow signal [67]. Improved visualization of the CC has also been achieved by multiple en face averaging of OCTA, leading to improved demarcation of capillary walls with a better delineation of intervascular spaces [68]. This technique has the potential to improve quantification methods of the CC. Further advances in OCTA imaging techniques will improve visualization and assessment of the CC. Currently, despite its limitations, OCTA has provided much valuable insight into CC alterations associated with dry AMD.

## Conclusions

It remains unclear whether hypoxia induced by CC flow impairment plays a predominant role in a mutually exclusive way or has a symbiotic link in the pathogenesis of AMD. Indeed, studies have suggested that molecular mechanisms within the RPE and photoreceptors associated with aging and stimulated by genetic or environmental factors also play a role. While much remains to be determined regarding the role of the CC in dry AMD, OCTA holds great promise in furthering our understanding of the pathophysiology of dry AMD, monitoring its progression, and assessing treatment.
